# Topical corticosteroid for treatment of hand osteoarthritis: study protocol for a randomised controlled trial

**DOI:** 10.1186/s12891-021-04921-2

**Published:** 2021-12-13

**Authors:** Yuanyuan Wang, Sultana Monira Hussain, Desmond Gan, Yuan Z. Lim, Mahnuma Mahfuz Estee, Stephane Heritier, Anita E. Wluka, Flavia M. Cicuttini

**Affiliations:** 1 School of Public Health and Preventive Medicine, Monash University, 553 St Kilda Road, Melbourne, VIC 3004 Australia; 2 Alfred Hospital, Melbourne, VIC 3004 Australia

**Keywords:** Topical corticosteroid, Osteoarthritis, Hand, Pain, Function

## Abstract

**Background:**

Hand osteoarthritis is a common and disabling chronic joint disease with a lack of effective therapies. Emerging evidence suggests the role of local inflammation in causing pain in hand osteoarthritis. Corticosteroids are potent anti-inflammatory drugs used in many rheumatic diseases. The aim of this randomised, double-blind, placebo-controlled trial is to determine whether topical corticosteroid reduces pain over 6 weeks in patients with hand osteoarthritis.

**Methods:**

One hundred participants with hand osteoarthritis will be recruited from the community in Melbourne, Australia, and randomly allocated in a 1:1 ratio to receive either topical Diprosone OV or placebo ointment administered 3 times daily on the painful hand joints for 6 weeks. The primary outcome is pain reduction (assessed by 100 mm visual analogue scale) at 6 weeks. The secondary outcomes include changes in pain and function assessed using Functional Index for Hand Osteoarthritis, Australian Canadian Osteoarthritis Hand Index, Michigan Hand Outcomes Questionnaire, and tender and swollen joint count at 6 weeks. Adverse events will be recorded. The primary analysis will be by intention to treat, including all participants in their randomised groups.

**Discussion:**

This study will provide high-quality evidence to determine whether topical corticosteroid reduces pain over 6 weeks in patients with hand osteoarthritis, with major clinical and public health importance by informing clinical practice guidelines for the management of hand osteoarthritis and reducing the burden of the disabling disease.

**Trial registration:**

Australian New Zealand Clinical Trials Registry (ANZCTR), ACTRN12620000599976. Registered 22 May 2020.

**Supplementary Information:**

The online version contains supplementary material available at 10.1186/s12891-021-04921-2.

## Background

Hand osteoarthritis (OA) is a common chronic joint disease causing pain, functional disability, and decreased quality of life which result in substantial burden of disease [1, 2]. The lifetime risk of symptomatic hand OA is 39.8% in the general population, 47.2% for females and 24.6% for males [3]. In people aged 55 years and over, 67% of the women and 54.8% of the men have radiographic OA in at least one hand joint, with 20% having disabling pain [4]. Hand OA impairs the activities of daily living such as dressing and eating [5], with significant clinical importance in relation to health-related quality of life [2]. The healthcare costs for treating this condition are expected to escalate due to the ageing population and increasing obesity [6]. Disease-modifying drugs for hand OA are currently not available. In terms of clinical guidelines for the management of hand OA, the European League Against Rheumatism suggests topical therapy (non-steroidal anti-inflammatory drugs (NSAIDs) being first-line choice), oral analgesics (e.g. paracetamol and NSAIDs), and non-pharmacological therapy (education, assistive devices, exercises and orthoses) [7]. The American College of Rheumatology conditionally recommends management with topical capsaicin, topical NSAIDs, oral NSAIDs, as well as non-pharmacological therapy [8]. Although there is some evidence for at best, a short-term moderate effect of topical and oral NSAIDs on pain alleviation, evidence is conflicting for the efficacy of anti-inflammatory medications, such as corticosteroids and biologic agents [9]. There is an unmet need for effective therapies for hand OA.

Increasing evidence suggests that hand OA is a disease of the whole joint with local inflammation playing an important role in causing pain and disease progression [1]. Imaging studies have shown that most patients with hand OA have synovitis (~ 50%) [10, 11], and that painful joints are more likely to have synovitis than non-painful joints [10, 12, 13]. Moreover, synovitis has been shown to be the strongest predictor for progression of structural damage in hand OA [14–17]. Therefore, inflammation is a potential treatment target in hand OA, and therapies targeting synovitis may provide a novel approach for the management of hand OA. Glucocorticoids are potent multitargeted anti-inflammatory drugs which have been used in many rheumatic diseases because of their anti-inflammatory and immunosuppressive actions [18]. Randomised controlled trials have shown the effect of oral corticosteroids (prednisolone) on reducing pain and improving function in patients with hand OA over 6 weeks [19, 20], with little effect over 3 months [19, 21]. However, systemic corticosteroids are associated with significant adverse events including diabetes and osteoporosis [22, 23]. Intra-articular injections of corticosteroids are effective but the effects often last less than 1 month [24]. It is also technically difficult to perform in small hand joints so often performed under ultrasound guidance which adds significantly to the cost, inconvenience and timeliness of treatment. Topical delivery of corticosteroids provides a potential alternative approach to improving outcomes in hand OA. Topical corticosteroids are safe, inexpensive and commonly used for skin conditions. However, there have been no clinical trials examining the efficacy of topical corticosteroids in reducing pain in hand OA.

### Objectives and hypothesis

We propose a randomised, double-blind, placebo-controlled trial to determine the effect of topical Diprosone OV ointment administered 3 times daily on painful hand joints compared to placebo in reducing pain and improving function in participants with hand OA over 6 weeks. It was hypothesised that topical Diprosone OV ointment would be more effective than placebo in reducing pain and improving function in participants with hand OA over 6 weeks.

### Study design

This is a single center, parallel-group, randomised, double-blind, placebo-controlled trial over 6 weeks.

### Trial registration

The trial was registered at the Australian New Zealand Clinical Trials Registry prior to recruitment commencing (ACTRN12620000599976, registered 22 May 2020). The trial reporting will be guided by the Consolidated Standards of Reporting Trials (CONSORT) Statement [25]. Protocol reporting is in accordance with the SPIRIT Statement (Standard Protocol Items: Recommendations for Interventional Trials) [26].

### Ethics approval

Ethics approval has been obtained from Alfred Hospital Ethics Committee (117/20) and registered at Monash University Human Research Ethics Committee (24219). Written informed consent will be obtained from all the participants.

## Methods

### Study setting and participants

Eligible participants with hand OA will be recruited from the community in Melbourne, Australia via advertisements and from medical practitioners.

#### Inclusion criteria

Participants aged ≥40 years with symptomatic radiological hand OA will be recruited. We will also invite those who contacted us to take part in the clinical trial of methotrexate for hand OA but could not take part due to concern about using methotrexate on the part of the participants or contraindications to its use. As recommended by the Osteoarthritis Research Society International (OARSI) clinical trials guidelines for hand OA [27], a pain score of at least 40 on a 100 mm visual analogue scale (VAS) and radiologic OA (Kellgren and Lawrence grade ≥ 2) in ≥1 hand joint will be required for study entry. Additional inclusion criteria include stable analgesic requirements (including NSAIDs) for at least 4 weeks, stable doses of chondroitin or glucosamine for 4 months, no corticosteroids via any route for at least 3 months.

#### Exclusion criteria

(i) concomitant rheumatic disease, inflammatory joint disease, psoriatic arthritis, ankylosing spondylitis, or gout; (ii) allergic reaction to other medicines containing betamethasone dipropionate, any other corticosteroid(s), or any of the ingredients of Diprosone OV ointment (i.e. betamethasone as dipropionate, propylene glycol, white beeswax, propylene glycol monostearate, and soft white paraffin); (iii) contraindication to topical corticosteroids (e.g. untreated bacterial, fungal, or viral skin lesions; widespread plaque psoriasis; skin conditions with ulcers); (iv) unable to complete informed consent; (v) pregnancy, breast feeding, or trying to become pregnant.

### Study timeline

**Fig. 1:**
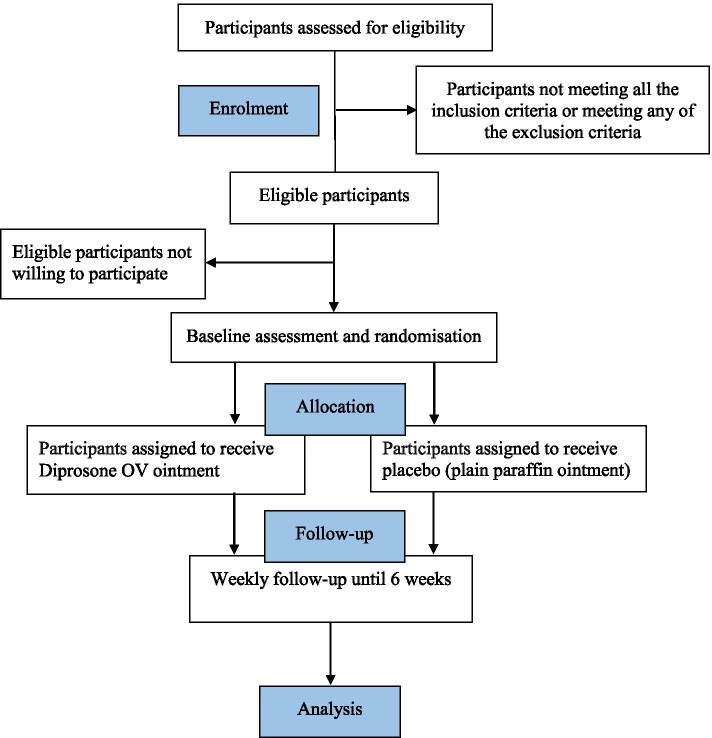
Flowchart of trial participation

This trial began recruitment in October 2020. Recruitment is estimated to be completed in January 2022 with the study finalized by 6-week follow-up and data collection in March 2022. Figure 1 shows trial participation and study procedure.

### Randomisation, allocation concealment, and blinding

Allocation of participants in a 1:1 ratio to either topical corticosteroid or placebo group will be based on computer generated random numbers prepared and securely held by the Alfred Health Clinical Trials Pharmacy. Block randomisation will be performed, stratified by gender given the higher prevalence of hand OA in females compared with males [4]. The use of a central automated allocation procedure with security in place will ensure the allocation cannot be accessed or influenced by any person. Participants, assessors and statisticians will be blinded to group allocation. Allocation concealment and blinding will be ensured by: (1) study medication being dispensed by the Alfred Health Clinical Trials Pharmacy; (2) use of an identical placebo ointment; (3) subjective measures being taken by assessors blinded to group allocation. Emergency unblinding will be allowed in limited situations that impact on the safety of study participants. Code-break for the full randomisation schedule will be maintained by the Alfred Health Clinical Trials Pharmacy, and be used in case of the need for emergency unblinding. Participants who are unblinded will be withdrawn from treatment and be requested to complete questionnaires at 6 weeks.

### Intervention

Participants will be randomised to receive topical corticosteroid or placebo. Placebo will be of the same colour and appearance as the corticosteroid ointment. They are required to apply a thin layer of the study ointment to cover the painful hand joints 3 times per day for 6 weeks.

#### Corticosteroid

Diprosone OV (betamethasone dipropionate in Optimised Vehicle) ointment. Diprosone OV contains the active ingredient betamethasone dipropionate which is a potent corticosteroid used to decrease inflammation, redness, itchiness and discomfort of some skin conditions. Diprosone OV ointment is used to treat persistent or severe dermatitis, eczema, and acute or chronic psoriasis.

#### Placebo

Plain paraffin ointment.

#### Safety

Adverse events will be recorded throughout the study. Photos of the hands, back and front, will be taken at baseline and 6 weeks to be reviewed by a dermatologist for safety check, mainly looking for dilatation of vessels, skin wrinkling, and hypopigmentation. Photos at 3 weeks will be taken and reviewed if there is any concern.

#### Compliance

The remaining ointment will be weighed at the end of the study to document compliance. Weekly telephone contact over the 6 weeks will be conducted to address any concerns. This will help to mitigate non-compliance.

#### Concomitant medication

To maintain the pragmatic nature of the trial, there are no restrictions about the use of concomitant analgesic medications. Participants will be allowed to continue the treatments being taken at the screening visit for the duration of the trial. If a participant is experiencing severe pain and requires an increase in analgesic dose, the use of paracetamol, topical or oral NSAIDs or opioids, or a combination of these will be permitted, with the reason for the dose increase and the dose used to be documented.

### Study procedure

Table 1 shows the study procedure. Volunteers will be telephone screened and undergo an x-ray of the symptomatic hand (standardized posteroanterior view) to confirm radiologic disease. In the case of bilateral symptomatic hands, the most symptomatic hand will undergo x-ray. In the case of both hands being symptomatic to an equal degree, the dominant hand will undergo x-ray. The study visits will be onsite or via telehealth. Consent will be obtained by the study doctor. At baseline, all the hand joints with a pain level of ≥40 on a 100 mm VAS will be documented on a hand map. The participants will be given a copy of this for reference when they are contacted by study staff by telephone. Participant will be asked to apply the study ointment on each of the pre-specified joints 3 times per day for 6 weeks. Participants are able to withdraw at any time during the trial; the time and reasons will be recorded. They will be requested to complete the questionnaires at 6 weeks.

**Table 1:** Timetable and measures to be made

	Screening	Double-blind period						
	Screening / baseline assessment	Randomization	Week 1	Week 2	Week 3	Week 4	Week 5	Week 6
**Visit/phone contact**	**0**	**1**	**2**	**3**	**4**	**5**	**6**	**7**
**Informed consent**	X							
**Clinical visit or telehealth**	X							X
**Telephone follow-up**			X	X	X	X	X	
**Hand x-ray**	X							
**Medical history**	X							
**Medication**	X							
**Employment and education**	X							
**Smoking and alcohol**	X							
**Questionnaires**								
Hand VAS	X							X
Hand NRS	X		X	X	X	X	X	X
FIHOA	X							X
AUSCAN	X							X
MHQ	X							X
painDETECT	X							X
**Physical examination**								
Height, weight	X							
Tender/swollen joint count	X							X
**Compliance and safety (adverse events)**			X	X	X	X	X	X
**Hand photos**	X							X
**Dispense medication**		X						

*VAS* Visual analogue scale, *NRS* Numerical rating scale, *FIHOA* Functional Index for Hand Osteoarthritis, *AUSCAN* Australian Canadian Osteoarthritis Hand Index, *MHQ* Michigan Hand Outcomes Questionnaire

### Primary outcome

Pain reduction at 6 weeks will be measured according to the OARSI recommendations for the design and conduct of clinical trials for hand OA, which recommend the use of a single question pain VAS as the main outcome measure for hand pain [27]. VAS has been most frequently used for pain assessment in hand OA, with excellent reliability, good construct validity and sensitivity to change [28].

### Secondary outcomes

#### Pain over 6 weeks

Participant will be asked weekly using a numerical rating scale (NRS) about their pain levels in general as well as in each of the study joints by referencing the hand map that they will have. Time to respond will be recorded.

#### Change in pain and function at 6 weeks

At baseline and 6 weeks, hand pain, physical function, and joint activity will be measured according to the OARSI recommendations [27] using the Functional Index for Hand OA (FIHOA) [29], Australian Canadian Osteoarthritis Hand Index (AUSCAN) [30], Michigan Hand Outcomes Questionnaire (MHQ) [31], and tender and swollen joint count [32].

### Other measures

#### Success of participant blinding

Participants will be asked which treatment they believe they received.

#### Treatment adherence

We will weigh the ointment container before and after the study.

#### Adverse events, analgesic use, and co-interventions

These will be measured in a log-book and by structured questioning by the blinded assessor at each follow-up.

#### painDETECT

*painDETECT*, a validated questionnaire used to assess pain sensitization in OA [33] will be assessed at baseline and 6 weeks.

#### Descriptive data

Self-reported age, gender, height, weight, duration of symptoms, employment, medical history, medication use, education level, smoking, and alcohol will be collected using a questionnaire.

### Sample size calculation

The mean VAS pain in a previous clinical trial of symptomatic hand OA similar to the proposed study was 54 mm (standard deviation 20 mm) [19]. The minimal clinically important difference to be detected in OA trials is a 15 mm change in VAS pain (out of 100 mm) [34]. We will require 40 participants per group to attain a power of 90% to detect the minimal clinically important difference (alpha 0.05, two-sided significance). Accounting for an estimated 20% loss to follow-up, we seek to recruit 100 participants, 50 in each group.

### Statistical analyses

An intention-to-treat analysis, including all participants in their randomised groups regardless of their adherence to assigned treatments, will be performed in a blinded fashion. Pain reduction at 6 weeks will be analyzed using ANCOVA with adjustment for the baseline measure. The bootstrap approach will be used to provide 95% confidence intervals and *p*-values if the normality assumption is questionable. Differences between randomised groups in trajectories of pain over time will be examined using linear mixed-effects regression models with baseline score as the covariate, fixed factors for treatment, time, and treatment x time interaction, and with an autoregressive AR (1) covariance matrix for repeated measures of individuals over time. Time to respond (weeks) will be compared between randomised groups. If more than 5% of participants are missing their primary outcome, multiple imputation will be applied to account for missing data, with missing values imputed separately by treatment group, assuming data are missing at random. Additional adjustment for age, gender, body mass index, and severity of radiographic OA will be carried out in a sensitivity analysis if clinically important baseline imbalances between randomised groups are identified. Pre-specified subgroup analyses will be performed by testing a treatment by subgroup interaction in the models. This will include joint group, radiographic severity, pain level, and presence of central sensitization. Per-protocol analyses for the primary and secondary endpoints will be conducted as secondary analyses.

### Data integrity and management

All data collected will be kept strictly confidential. Data will be collected using prespecified case report forms in REDCap. Paper copies of questionnaires (if participants prefer to complete the questionnaires on hard copy) will be stored in locked filing cabinets, with secured and restricted access. Electronic data will be stored in REDCap, and exported to password-protected servers with secured and restricted access after data collection, separating the identifying and non-identifying information. In the dataset, participants will be identified by study ID and randomisation code, with other information with the potential of identifying individuals removed. The codes linking data to identifying participant information will be kept separately from the study data, under password protection and with restricted access. After trial completion, case report forms will be securely archived, and electronic data will be saved on the secure server, password-protected and accessible only to study investigators. There are no planned interim analyses and stopping guidelines.

This study uses REDCap for data collection, facilitating telehealth options. For participants who use the telehealth option for the screening/baseline visit, we will seek consent electronically (eConsent). REDCap has a feature that implements consent forms through an online survey which can be accessed on a computer, mobile phone, or tablet. The completed eConsent PDFs are stored in REDCap in a File Repository under “PDF Survey Archive”.

### Monitoring

The study investigators will monitor the study execution, ensuring that trial execution is consistent with the study protocol. Regular meetings will ensure efficient study completion and monitoring of adverse events. Study investigators will perform annual self-auditing of study execution according to the School of Public Health and Preventive Medicine, Monash University. We will not need a data safety monitoring board as this agent is Therapeutic Goods Administration approved with well-known safety profile.

### Dissemination

Trial results, regardless of statistical significance, will be published in peer-reviewed journals and presented at national and international conferences. Upon publication of the primary manuscript, participants will be informed of their group allocation and provided with the results.

## Discussion

This randomised controlled trial is conducted to determine whether topical Diprosone OV ointment administered 3 times daily reduces pain over 6 weeks in participants with symptomatic hand OA.

Synovial pathology of hand OA has shown histological changes that are consistent with those in rheumatoid arthritis and manifest the stage of disease at the time of biopsy [35]. There are increased levels of pro-inflammatory cytokines, reduced levels of anti-inflammatory cytokines, infiltration of mononuclear cells and adaptive immune cell responses within OA fluid and tissues [36–38]. Local inflammation has been shown to play a role in causing pain and disease progression in hand OA [1], suggesting inflammation could be a potential treatment target. Randomised controlled trials have demonstrated that oral and intra-articular injections of corticosteroids may reduce pain over short-term in hand OA [19, 20, 24], providing proof of concept that targeting inflammation in hand OA is effective for pain relief. However, there is the concern of significant adverse events associated with systemic corticosteroids [22, 23]. Topical corticosteroid would provide a safe, alternative therapeutic approach to reducing pain in hand OA, which will be tested in the proposed clinical trial.

This study will provide high-quality evidence to determine whether topical corticosteroid reduces pain over 6 weeks in patients with hand OA, with major clinical and public health importance by informing clinical practice guidelines for a potentially effective treatment option for hand OA and reducing the burden of the disabling disease.

## Supplementary Information

**Additional file 1.**

## Data Availability

Data sharing is not applicable to this article as no datasets were generated or analysed during the current study.

## Abbreviations

AUSCAN: Australian Canadian Osteoarthritis Hand Index
CONSORT: Consolidated Standards of Reporting Trials
FIHOA: Functional Index for Hand Osteoarthritis
MHQ: Michigan Hand Outcomes Questionnaire
NRS: Numerical rating scale
NSAIDs: Non-steroidal anti-inflammatory drugs
OA: Osteoarthritis
OARSI: Osteoarthritis Research Society International
VAS: Visual analogue scale
